# Atrial fibrillation associated with heart failure treated by a 2-lead CRT-DX system (BIO-AffectDX): Study design and clinical protocol

**DOI:** 10.1016/j.hroo.2021.10.001

**Published:** 2021-10-09

**Authors:** Jonathan C. Hsu, Aaron B. Hesselson, Jackson J. Liang, Stavros Mountantonakis, Gregory T. David, Alexandru Costea

**Affiliations:** ∗ Cardiac Electrophysiology Section, Division of Cardiology, Department of Medicine, University of California, San Diego, La Jolla, California; † University of Kentucky Gill Heart & Vascular Institute, Lexington, Kentucky; ‡ Electrophysiology, Division of Cardiology, Internal Medicine, University of Michigan, Ann Arbor, Michigan; § Division of Cardiac Electrophysiology, Lenox Hill Hospital, Northwell Health, New York, New York; ‖ BIOTRONIK, Inc, Lake Oswego, Oregon; ¶ Clinical Cardiac Electrophysiology at University of Cincinnati Medical Center, Cincinnati, Ohio

**Keywords:** Atrial fibrillation, Cardiac resynchronization therapy, CRT-DX, Heart failure, Two-lead CRT-D

## Abstract

**Background:**

Evidence to support use of cardiac resynchronization therapy (CRT) among patients with both heart failure (HF) and atrial fibrillation (AF) is largely limited to retrospective or post hoc subanalyses. Data from a prospectively enrolled and contemporary cohort are needed.

**Objective:**

We aim to better characterize the changes from baseline in HF patients with concomitant AF subsequently implanted with a 2-lead CRT-DX system capable of sensing in the atrium, aggregating diagnostics, and delivering CRT therapy. The primary objective of this study is to evaluate the percentage of all HF subjects with an improvement in a clinical composite score from pre-CRT implant to 12 months.

**Methods:**

The study is a US-based, prospective, observational multicenter clinical trial conducted at up to 50 sites and enrolling approximately 400 subjects with a follow-up period of 1 year. Multiple subject assessments, atrial rhythm status, and device interrogation will be collected at follow-up visits occurring at 3, 6, and 12 months postimplant.

**Results:**

A Clinical Events Committee will adjudicate subject HF events, arrhythmia events, death events, and all device-classified ventricular tachycardia and ventricular fibrillation episodes with treatment that are collected throughout the follow-up period. Their decisions are based on independent physician review of the data from sites and device interrogation.

**Conclusion:**

The BIO-AffectDX study aims to provide further insight into the expected outcomes from CRT treatment in patients with HF and AF.

**Key Findings**

▪ The findings from the BIO-AffectDX study will help address knowledge gaps of cardiac resynchronization therapy (CRT) utilization in concurrent heart failure and atrial fibrillation (AF). With a specific focus on differences between AF subtypes, this prospective study evaluates the effectiveness of CRT in patients that have a standard CRT-D indication per current guidelines.
▪ The BIO-AffectDX study utilizes a clinical composite score to evaluate CRT effectiveness after 1 year of follow-up as compared to baseline prior to device implant. The study also aims to collect information to further understand the association between ablation for atrial fibrillation and CRT.
▪ The study utilizes a novel 2-lead CRT system that provides atrial sensing diagnostics, which allows for monitoring of atrial status. Atrial status will be correlated with procedures that may impact atrial rhythm, as well as spontaneous conversion events to further elucidate how these events impact CRT effectiveness.
▪ The results of the BIO-AffectDX study are expected to inform future societal consensus and guidance documents regarding the value of CRT in patients with both heart failure and atrial fibrillation.

## Introduction

### Prior studies of cardiac resynchronization therapy in atrial fibrillation patients

Heart failure (HF) and atrial fibrillation (AF) often coexist, with one condition occasionally predisposing the other. It is estimated that approximately 20% of patients with HF, and 25% of patients receiving cardiac resynchronization therapy (CRT), have some form of AF.^1^^,^^2^ CRT is a proven treatment shown to improve quality of life, functional status, and mortality in an indicated HF population.^3^, ^4^, ^5^ However, many early clinical trials evaluating the effectiveness of CRT excluded patients with AF, making it difficult to ascertain a benefit in this specific comorbid population. As a result, current guideline-concordant indications call for CRT in patients with AF as a class IIa level of evidence B, while patients with sinus rhythm are a class I level of evidence A indication.^6^

CRT effectiveness in patients with HF and AF has been evaluated in a limited number of studies with conflicting results.^2^^,^^3^^,^^7^, ^8^, ^9^ Interpretation of the existing data has relied heavily on secondary analyses of subgroups from randomized controlled trials and retrospective analyses of national registries. Furthermore, there is some limited evidence suggesting that CRT-D may reduce the frequency and duration of AF in patients with HF.^10^^,^^11^ More prospective data are needed to corroborate these findings.

Additionally, in a 2020 review of CRT research a panel of experts examined the existing clinical data related to CRT effectiveness in patients with HF with reduced ejection fraction and identified several evidence gaps related to CRT.^12^ Two of the top-tier gaps related to the effectiveness of CRT among patients with paroxysmal, persistent, and permanent AF and the association and role of atrioventricular (AV) node ablation on CRT effectiveness.

### Role of atrial lead in CRT

Since 2006, studies have consistently supported the concept that higher percentages of biventricular pacing are associated with improved patient outcomes, with optimal percentages ranging from 90% up to 100%.^13^, ^14^, ^15^, ^16^ Effective CRT pacing is difficult to achieve in the setting of AF, as loss of AV synchrony, uncontrolled ventricular rates, and a high percentage of fusion and pseudo-fusion beats can contribute to suboptimal delivery of CRT pacing.^17^ Traditional CRT-D systems have utilized 3 leads historically (atrial, right ventricular, and left ventricular [LV]). When a patient is indicated for atrial pacing support it is self-evident that an atrial lead is warranted and appropriate. This lead may also provide the added benefit of improving supraventricular and ventricular arrhythmias along with identifying atrial high-rate episodes. Additionally, implanters may prefer atrial placement at the time of CRT-D implantation to avoid a reoperation in the event a patient develops the need for pacing support at a later time. The decision to implant an atrial lead in patients without a need of atrial support does not currently reflect practices in the United States. Wide regional variation exists in the United States regarding when to place an atrial lead and there is evidence with implantable cardioverter-defibrillators (ICDs), at least, that nearly two-thirds of patients without a pacing indication are implanted with an atrial lead.^18^ The added procedural and long-term risks associated with this third lead are not insignificant, as studies have identified increased rates of periprocedural complications, in-hospital mortality, and complications within 90 days of device implantation.^19^ Additionally, it has been observed that implantation of atrial leads can be detrimental in that they can actually promote AF.^20^ These added risks combined present a challenge for current physicians considering the risk-benefit ratio of an atrial lead. Furthermore, the utilization of atrial pacing specifically in CRT patients without a clear need for atrial pacing is not well established.^21^ Additionally, the 2012 EHRA/HRS expert consensus statement on cardiac resynchronization therapy recommended using VDD/DDD mode with a base rate of 35–40 beats per minute to ensure permanent or nearly permanent atrial sensing and avoid atrial support altogether.^22^

### Two-lead CRT system

BIOTRONIK’s CRT-DX system can deliver CRT utilizing a novel 2-lead system that incorporates atrial sensing on the ICD lead for supraventricular tachycardia discrimination and AV synchronization, thus allowing for CRT delivery with fewer leads. The 2-lead CRT-DX systems are considered for patients without a need for atrial pacing, including those with permanent AF, and may preserve the benefits of sensing atrial activity without the need to implant an atrial lead.

Although experience with this system is limited, the emerging data are encouraging and support the safety of this system in carefully selected patients. Biffi and colleagues^23^ compared clinical and technical outcomes of 25 CRT-D recipients with 3-lead systems and 12 patients implanted with a CRT-DX. An absolute indication for atrial stimulation was observed in 1.2% of CRT recipients during a 3-year follow-up period. CRT response and chronotropic incompetence was evaluated and there was no difference between the 2 groups observed with respect to NYHA class improvement, peak cardiopulmonary performance, reverse remodeling, and the presence of chronotropic incompetence at 1 year. There were also no patients observed to develop a need for atrial pacing support at 3 years with an absence of atrial undersensing. Additionally, a recent subanalysis of 240 patients demonstrated significantly fewer complications and lower rates of inappropriate shock among patients implanted with a CRT-DX system while supporting similar health status outcomes as compared to patients implanted with a conventional 3-lead CRT system.^24^ However, it is difficult to extrapolate these findings to patients with AF, since AF history and ablation status within each cohort were unknown.

There is also some evidence that suggests patients in AF receiving CRT may experience a reduction in overall atrial burden and up to 10% of patients may spontaneously convert back to sinus rhythm.^25^ The CRT-DX system may offer an added benefit of providing appropriate detection of AF and mode switch capability and provide ventricular pacing support during pauses without the need and increased risk that is associated with an atrial lead. Compared to systems without a dedicated atrial lead, DX has been proven superior with regard to detection of atrial tachycardia / AF, which allows for earlier intervention.^23^^,^^26^ Based on the need for additional prospective data, the BIO-AffectDX Study is designed to evaluate the percent of subjects with 12-month improvement from pre-CRT implant in a large cohort of HF patients with AF implanted with a novel, 2-lead CRT system. The analysis particularly emphasizes a comparison of patient outcomes between the AF subtypes.

## Objectives

The primary objective of this study is to evaluate the percentage of all HF subjects with an improvement in a clinical composite score (CCS) from pre-CRT implant to 12 months. Subjects will be stratified by paroxysmal, persistent, and long-standing persistent AF subtypes implanted with a 2-lead CRT-DX system.

Secondary objectives include the following: (1) to evaluate the rate of composite all-cause death and HF hospitalizations through 12 months; (2) to compare AF subtypes for CCS status, changes in Atrial Fibrillation Effect on Quality-of-Life Questionnaire, changes in Minnesota Living with Heart Failure Questionnaire, changes in NYHA, changes in 6-minute walk test, and changes in AF burden at 6 and 12 months; (3) to evaluate the proportion of subjects showing improvement from pre-CRT using a CCS at 6 months; (4) to evaluate the observed major complication rates from device implantation in this population at 12 months; (5) to evaluate the percentage of subjects converting to sinus rhythm at any time during the 12-month follow-up period, along with reason for conversion, both overall and by AF subtype.

## Design

### General considerations

The BIO-AffectDX Study is a multicenter, prospective, observational, single-arm study designed to evaluate the rate of subjects showing improvement from pre-CRT using a CCS at 12 months in up to 400 HF subjects with paroxysmal, persistent, or long-standing persistent AF subtypes and a 2-lead CRT-DX system. This study is an industry-initiated clinical trial designed by a steering committee consisting of physicians (who also serve as investigators) in collaboration with the sponsor. Study subjects will be enrolled at up to 50 participating US sites. Target enrollment percentages are 50%, 30%, and 20% of the total study population for paroxysmal, persistent, and long-standing persistent AF, respectively. Patients will be screened for the inclusion and exclusion criteria and sign an informed consent prior to baseline assessments, which include quality-of-life assessment, 6-minute walk test, NYHA class assessment, rhythm collection, and pertinent medical history. Subjects will be implanted with a CRT-DX (BIOTRONIK SE & Co KG, Berlin, Germany) pulse generator and DX ICD (BIOTRONIK SE & Co KG) lead, plus an LV lead within 30 days following informed consent. All subjects eligible for the study will receive a new (de novo) implant or undergo an upgrade from an existing single-chamber ICD system that includes a DX ICD lead. Subjects will be seen for follow-up visits at 3, 6, and 12 months postimplant. In addition, details on ablation or cardioversion procedures occurring during follow-up will be collected, which include recording atrial rhythm pre- and postprocedure, the cardioversion and catheter ablation procedure date, type and success as determined by the investigator, and device programming and settings, as well as device diagnostics, electrical parameters, and programmed parameters. HF, all-cause mortality, and arrhythmia events will be collected and adjudicated by independent event committees. All-cause death and HF hospitalization among all subjects will be calculated as raw number and percentage of subjects at 12 months. Mortality will be assessed by each site by a thorough review utilizing all available medical records. HF events will include HF hospitalization and worsening HF events without hospitalization. Adjudicated arrhythmic events will be limited to device-classified ventricular tachycardia (VT) and ventricular fibrillation (VF) episodes with shock treatment. Device-classified VT and VF episodes with antitachycardia pacing but no shock that transmitted and are available on home monitoring will also be adjudicated. Study results will be hypothesis generating, given the observational design of the study without use of a prospective control arm.

### Sample size justification

There is no formal inferential analysis scheduled for the primary objective. The study was designed to limit the number of subjects involved, while still providing CRT-DX therapy to a sufficiently large subject population to ensure a representative and statistically meaningful sample. It is anticipated that at least 400 subjects will be implanted and followed through 12 months postimplantation at up to 50 US sites. The sample size required to assess the primary objective is based on the precision of improvement from baseline achieved using an exact point estimate and 1-sided upper 95% confidence interval. The sample size for the primary objective was calculated based on the following assumptions:

The standard normal approximation to the 95% confidence interval associated with an estimate of a binomial parameter (p) is given by:

95% Confidence Interval: p ± 1.96 [ p (1 − p) / n ]^1/2^

To meet the enrollment goals for the BIO-AffectDX study, target enrollment percentages within each AF subtype and based on prior use of CRT-DX systems are defined as 50%, 30%, and 20% of the total study population for paroxysmal, persistent, and long-standing persistent AF, respectively. Therefore, it is expected that no single AF subtype will be greater than 60% of the total study population.

A limitation of using the above method is that secondary and subanalyses are exploratory and will not be adequately powered. This will be mentioned in all results, with confidence intervals and effect sizes also given to provide complete transparency, with a clear distinction that owing to small samples, the results likely overestimate the true effect size in the population.

### Eligibility

Subjects will be eligible for enrollment if they meet the standard CRT-D indication according to current guidelines and have a documented history of paroxysmal, persistent, or long-standing persistent AF. Paroxysmal AF will be defined as AF that terminates spontaneously, or with intervention within 7 days. Persistent-continuous AF will be defined as sustained AF beyond 7 days. Long-standing persistent-continuous AF will be defined as AF of greater than 12 months in duration. The full list of eligibility criteria is summarized in Table 1.

**Table 1:** Study eligibility criteria

Inclusion criteria	• Standard CRT-D indication according to current guidelines • Patient has documented history of paroxysmal, persistent, or long-standing persistent AF • De novo implant or upgrade from a DX ICD system • Implant planned to occur no more than 30 days after signing consent • Patient is able to understand English or Spanish • Patient is able to understand the nature of the study and provide informed consent • Patient is willing and able to complete all routine study visits at the investigational site for up to 12 months of follow-up • Patient is willing to utilize BIOTRONIK Home Monitoring® via CardioMessenger • Patient age is greater than or equal to 18 years or older
Inclusion criteria after consent has been obtained	• Baseline subject assessment is evaluated as NYHA class II, III, or ambulatory IV heart failure at study enrollment visit • Baseline subject assessment of 6-minute walk test is completed at study enrollment visit and walk distance ≤450 meters (1476 feet)
Exclusion criteria	• Contraindication to CRT-D/CRT-DX • Patient has current or previous atrial pacing need • Patient is considered for a His bundle pacing system • Patient has current or previous pacemaker, non-DX ICD implant, or biventricular pacing system prior to enrolling • Patient is currently planned for a PVI catheter ablation procedure within 3 months of consent • Patient life expectancy is less than 1 year • Patient is expected to receive heart transplantation or ventricular assist device within 1 year after implant

AF = atrial fibrillation; CRT = cardiac resynchronization therapy; ICD = implantable cardioverter-defibrillator; PVI = pulmonary vein isolation.

### Protection of human rights, recruitment, and consent

Institutional Review Board approval is required from each institution prior to participation in this clinical study. Written informed consent will be obtained from the patient prior to collection of any study-specific data.

Consented subjects will be considered provisionally enrolled until a CRT-DX is implanted successfully. Subjects with both signed informed consent and an implanted CRT-DX system are considered enrolled. Provisionally enrolled subjects that exit prior to implant or do not receive a CRT-DX system within 30 days of signing consent will be considered screen failures, and will not be included in the analysis population or count toward the 400-subject enrollment goal.

### Baseline data collection

**Figure 1:**
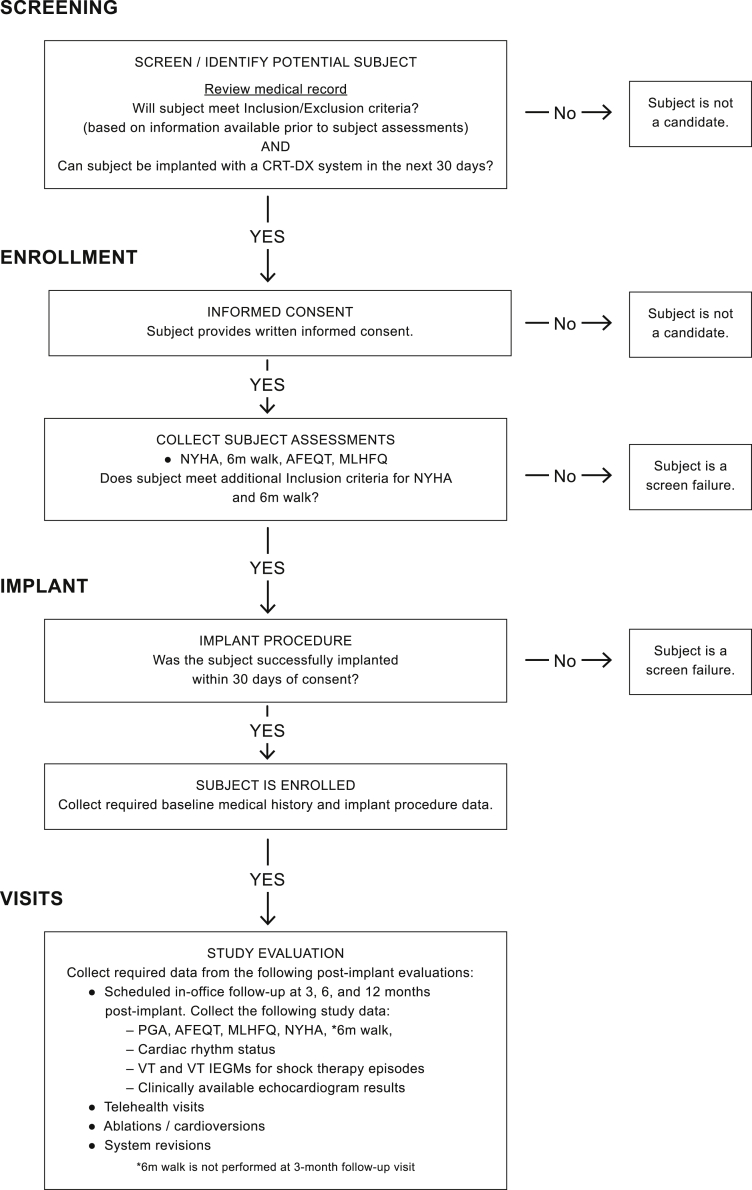
BIO-AffectDX Study design flowchart. AFEQT = Atrial Fibrillation Effect on Quality-of-Life Questionnaire; CRT = cardiac resynchronization therapy; IEGM = intracardiac electrogram; MLHFQ = Minnesota Living with Heart Failure Questionnaire; NYHA = New York Heart Association classification; PGA = Patient Global Assessment; VT = ventricular tachycardia.

Baseline data collection such as basic demographics, medical history, current cardiovascular and oral anticoagulant medications, AF subtype, ablation history, HF hospitalization, and arrhythmia hospitalization history will be collected for fully enrolled subjects. Electrocardiogram patch, Holter monitor, and recent echocardiogram results obtained ≤6 months prior to implant will be collected and analyzed if data are available. The study flowchart outlining screening, enrollment, implantation, and study visits of subjects in BIO-AffectDX is shown in Figure 1.

### Device implantation

Patients will be implanted with a CRT-DX system according to current clinical standards, based on current guideline-based clinical indications for a CRT-D. Implantation with a CRT-DX system can take place no more than 30 days after consent. The CRT-DX system will be determined by the site investigator to be a successful implantation of the CRT-DX pulse generator, a DX ICD lead, and a quadripolar LV lead. A CRT-DX system is considered “unsuccessful” if any component of the CRT-DX system (CRT-DX pulse generator, DX ICD lead, and LV lead) is attempted to be implanted but could not be placed in a stable position, or if the CRT-DX system is unable to deliver adequate therapy owing to any cause attributable to the implant procedure as determined by the investigator. Additionally, geographic location of the atrial dipole within the atrium will be assessed visually by the site investigators. The anatomic locations within the atrium will be characterized at the time of implant as upper third, mid third, lower third, or unknown. Lead implantation and implant approaches will be performed according to institutional standard procedures of the enrolling centers. Although it is not required by protocol, sites choosing to perform defibrillation threshold testing will do so according to their institutional standards.

### Device programming

In the BIO-AffectDX study the devices are programmed to achieve maximal biventricular pacing percentage; therefore, at a minimum, LV pacing is required to be programmed ON. The pulse generators in this study provide a wide range of mode settings and 20 different LV pacing vectors and configurations, including integrated bipolar LV pacing and true LV bipolar pacing. Additional programming options that are available include triggered pacing and timing options such as initially paced chamber and/or interventricular delay programming. The protocol allows for investigator judgment in determining these features for individual subjects. The percentage of CRT pacing will be determined and confirmed by analyzing device diagnostics from in-office interrogations and home monitoring transmissions. DX sensing, home monitoring transmission to allow collection and evaluation of daily remote data, and intracardiac electrogram collection for tachycardia therapy are to be programmed ON. Study subjects should be registered in the Home Monitoring Service Center as soon as possible following implant. Specific bradyarrhythmia settings, tachyarrhythmia settings, and CRT optimization are at the discretion of the investigator. Required and recommended settings for programmed parameters are provided in Table 2.

**Table 2:** Required and recommended programmed parameters

Parameters	Programmed setting
Ventricular pacing	BiV (required)
DX sensing	ON (required)
BIOTRONIK Home Monitoring®	ON (required)
IEGMs for therapy episodes	ON (required)
SMART Detection®	ON
ICD therapy	ON
MPP	OFF

ICD = implantable cardioverter-defibrillator; IEGM = intracardiac electrogram; MPP = multipole pacing.

### Follow-up

Following patient consent and the implantation procedure, subjects will be evaluated during in-office follow-up visits at 3, 6, and 12-month postimplant. Subject assessments at each visit will include an Atrial Fibrillation Effect on Quality of Life questionnaire, Minnesota Living with Heart Failure Questionnaire, and an NYHA class assessment. In addition, a Patient Global Assessment (PGA) will be given to collect the patient’s reported rating of overall health at each follow-up compared to prior to receiving the CRT system. Subject responses to the PGA are captured on a scale of 7 responses, from Markedly Better to Markedly Worse. Additionally, at the 6- and 12-month visits a 6-minute walk test will be administered. After completion of subject assessments, a routine device interrogation, review of all stored device-classified VT and VF shock intracardiac electrograms, and cardiovascular and oral anticoagulation medication reconciliation will occur. An evaluation of current atrial rhythm via a standard method (3-lead, 5-lead, 12-lead, programmer strip, etc) will also be collected. Echocardiography data will be obtained and analyzed for subjects with routine echocardiography testing data available both at baseline and 12-month follow-up. Some of the echocardiography data of interest will include left atrial size and LV dimensions and degree of mitral regurgitation in addition to other clinically relevant parameters. Adverse events including HF, arrhythmia, and death events will be collected at each visit.

Telehealth follow-up visits are replacing in-office follow-up visits in many centers. While the BIO-AffectDX Study strongly recommends an in-office visit whenever possible, telehealth visits in conjunction with a live phone or video call between the subject and investigational site staff are allowed. As conventional methods used to obtain a subject’s cardiac rhythm status are not available during a telehealth follow-up visit, an on-demand remote device data download will be used to provide current rhythm status for the subject.

### Ablation/cardioversion procedures

Ablation and cardioversion procedures occurring during the study will be documented. Specifically, atrial rhythm pre- and postprocedure, date and type of procedure, success of procedure, postprocedure device programming and settings, and any reportable adverse events will be obtained. Success of these procedures will be determined and specified by the site investigator based upon their clinical experience. Additionally, cardioversion from AF identified by site personnel to be the result of shocks delivered during the implantation procedure will be captured.

### Data analysis

For all study objectives and any additional data of interest collected, descriptive statistics will be used to present and summarize the data collected during the study.

Frequency distributions and cross-tabulations will be presented for discrete variables. Means, standard errors, and ranges will be presented for continuous variables.

The primary objective of this study is to evaluate the number and percentage of all HF patients with paroxysmal, persistent, and long-standing persistent AF subtypes showing improvement from baseline at 12 months. A CCS based on changes in NYHA class, PGA status, HF hospitalization status, and all-cause death is utilized to define the primary objective. The primary objective will be assessed by performing an exact point estimate and 1-sided upper 95% confidence interval for the rate of improvement.

**Figure 2:**
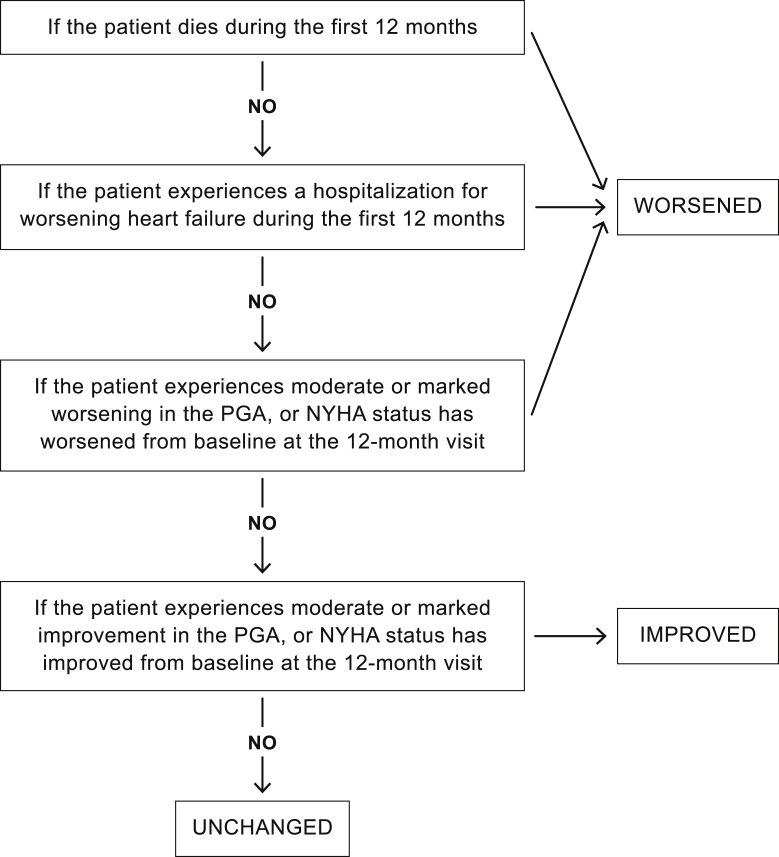
Hierarchy of clinical composite score. NYHA = New York Heart Association classification; PGA = Patient Global Assessment.

The CCS employs a hierarchical method to classify each subject with a status of “Improved,” “Worsened,” or “Unchanged.” A patient is classified as “Worsened” if they have experienced death due to any cause, a HF hospitalization, a moderate or marked worsening in PGA, or worsening of NYHA functional class compared to baseline. A patient is considered “Improved” if they improved in at least 1 of the NYHA or PGA and did not worsen in any component. All other patients are classified as “Unchanged.” The hierarchy to determine CCS classification is displayed in Figure 2. The PGA will ask subjects to assess how their overall status has changed since prior to receiving CRT therapy.^27^^,^^28^

In the BIO-AffectDX study, subjects will be analyzed on both an intention-to-treat and an as-treated basis to account for removal or exchange of the implanted CRT-DX device system. The primary analysis for the primary objective will include the entire cohort, including all AF subtypes. Secondary objective analysis for each AF subtype (ie, paroxysmal AF, persistent AF, and long-standing persistent AF) subgroup will utilize the subtype documented at the baseline medical history collection using the intention-to-treat population.

Several prespecified and preplanned exploratory subgroup analyses on primary and secondary objective data will also be performed, when it is determined feasible and appropriate based on the available data The prespecified subgroups may include, but are not limited to, the following: AF subtype, AF burden, age, sex, race, NYHA class, LV ejection fraction, type of cardiomyopathy, QRS type and duration, ablation type and status, LV lead position and pacing vector, DX lead tip position in addition to the atrial dipole position (based upon operator visual assessment as high atrium, mid atrium, low atrium, or other), number of hospitalizations within the last 12 months, number of HF or arrhythmia hospitalizations within the last 12 months, ventricular pacing percentage, left atrial size and LV dimensions obtained during baseline echocardiography, degree of mitral regurgitation, and clinically relevant echocardiographic data. Additionally, study objective outcomes may be compared to rates from published literature and/or outcomes from a contemporary control group of subjects in sinus rhythm receiving a CRT-D. Daily remote monitoring data, including but not limited to AF burden and ventricular arrhythmias, will be obtained throughout the study participation. A variety of analyses will be conducted on the subgroups depending on the structure of the data and given normality. For categorical data this includes χ^2^, binomial proportion test, and McNemar test, while for continuous data includes paired *t* test, *t* test, and 2-sample *t* test. If the assumption of normality is not met, then nonparametric versions of the appropriate method will be used. Effect sizes and 95% confidence intervals will be given for all results of the subanalyses. An alpha of 0.05 will be used, and all analysis will be conducted with SAS 9.4 (SAS Institute Inc, Cary, NC).

## Significance of the trial

The BIO-AffectDX study will provide important clinical information regarding improvement in clinical outcomes in patients with HF and AF who receive a CRT-D device. There are several knowledge deficits in the space targeted by the BIO-AffectDX study. First, since many prospective clinical trials investigating improvement of patients receiving CRT-D excluded patients with AF, the BIO-AffectDX study will evaluate the overall improvement of a CCS of HF in patients that have been identified as having evidence gaps related to the effectiveness of CRT, namely those with coexisting AF and HF. Patients enrolled in the BIO-AffectDX study will all have a history of AF. All subtypes of AF will be enrolled, including paroxysmal, persistent, and long-standing persistent AF. Given the exclusion of AF patients in many CRT trials, the BIO-AffectDX study will be one of the largest prospective studies to ever study clinical outcomes in this unique population. Second, additional secondary outcomes in patients with AF and congestive heart failure receiving CRT-D therapy will be evaluated in the BIO-AffectDX study, including quality of life, HF hospitalization, death, and maintenance of sinus rhythm. Therefore, the BIO-AffectDX study will be able to specifically analyze several clinical outcomes in this population of patients, and evaluate differences based on the patient subtype of AF. Third, the BIO-AffectDX will utilize a unique CRT-D system that does not require implantation of a separate atrial lead, but utilizes an atrial dipole contained within the high-voltage defibrillator lead for atrial sensing. The study allows for the evaluation of outcomes in patients with AF and HF that qualify for CRT-D therapy with the use of fewer leads to provide this CRT therapy and will provide important insights into outcomes in AF patients who qualify for CRT therapy but may not require atrial pacing therapy.

The authors anticipate that the results of this important study will inform future societal consensus and guideline documents that call for further study of the effect of CRT in AF patients, and will help clarify the role of CRT therapy in patients with both AF and HF that qualify for CRT therapy use.

## Funding Sources

This study is supported by BIOTRONIK, Inc.

## Disclosures

Jonathan Hsu has received honoraria from Abbott, Biosense-Webster, Biotronik, Boston Scientific, Bristol-Myers Squibb, Janssen Pharmaceuticals, Medtronic, Pfizer, and Zoll Medical, and research grants from Biosense-Webster and Biotronik, and has equity interest in Acutus Medical and Vektor Medical. Aaron Hesselson has received honoraria from Biotronik and Impulse Dynamics. Jackson Liang has received honoraria from Abbott, Biotronik, and Boston Scientific. Stavros Mountantonakis has received honoraria from Biotronik, Medtronic, and Zoll Medical and research grants from Abbott and Biotronik. Gregory David is a salaried employee of Biotronik, Inc. Alexandru Costea has received honoraria from Biosense-Webster and Biotronik and research grants from Biotronik.

## Authorship

All authors attest they meet the current ICMJE criteria for authorship.

## Patient Consent

Written informed consent will be obtained from the patient prior to collecting any study-specific data.

## Ethics Statement

Institutional Review Board approval is required from each institution prior to participation in this clinical study.
